# Down-Regulation of Proline Rich Homeodomain/Haematopoietically Expressed Homeobox Expression in Prostate Cells Enables Tumour Initiation and Tumour Growth

**DOI:** 10.3390/cancers18142247

**Published:** 2026-07-14

**Authors:** Eudmar Marcolino, Jinxia Zheng, Christopher Roberts, Eric Vancauwenberghe, Ahmed Alhajuji, Ian G. Mills, Abeer M. Shaaban, Sebastian Oltean, Padma-Sheela Jayaraman, Kevin Gaston

**Affiliations:** 1 School of Medicine and Biodiscovery Institute, University of Nottingham, Nottingham NG7 2RD, UKchristopher.roberts2@nottingham.ac.uk (C.R.); ahmed.alhajuji@nottingham.ac.uk (A.A.); 2 Department of Clinical and Biomedical Sciences, University of Exeter Medical School, Exeter EX1 2LU, UKs.oltean@exeter.ac.uk (S.O.); 3 Nuffield Department of Surgical Sciences, University of Oxford, Oxford OX3 9DU, UK; ian.mills@linacre.ox.ac.uk; 4 Johnston Centre for Cancer Research, Queen’s University Belfast, Belfast BT9 7AE, UK; 5 Department of Cancer and Genomic Sciences, University of Birmingham, Birmingham B15 2TT, UK; abeer.shaaban@uhb.nhs.uk; 6 Cellular Pathology, Queen Elizabeth Hospital Birmingham, Birmingham B15 2GW, UK

**Keywords:** prostate, tumour suppression, transcription, PRH/HHEX, Protein Kinase CK2

## Abstract

**Background:** The Proline Rich Homeodomain/Haematopoietically Expressed Homeobox (PRH/HHEX) transcription factor down-regulates the proliferation of prostate cells, and it has been suggested that this protein acts as a tumour suppressor in prostate epithelial cells. **Results:** Here, we show that the *HHEX* gene encoding PRH, located at chromosome 10q23, is often deleted in prostate cancer cells. Moreover, the gene encoding PRH displays increased CpG methylation in prostate cancer cells, and PRH mRNA levels and protein levels are decreased in high Gleason grade prostate tumours. Using a doxycycline-inducible model, we show that over-expression of PRH in prostate cancer cells reduces cell proliferation and cell migration in vitro and inhibits tumour growth and tumour initiation in a mouse xenograft model. Similarly, PRH over-expression in a syngeneic mouse model reduces tumour growth. Interestingly, the inhibition of Protein Kinase CK2 in this model results in increased PRH protein levels in vitro, decreased cell viability, and reduced tumour growth in vivo. **Conclusions:** PRH acts as a tumour suppressor protein in prostate cancer cells and the re-establishment of PRH activity in these cells through the inhibition of PRH phosphorylation, or through other means, could be a useful approach to prostate cancer treatment.

## Simple Summary

The Proline Rich Homeodomain/Haematopoietically Expressed Homeobox (PRH/HHEX) transcription factor inhibits the proliferation of prostate cells, and the levels and activity of this protein are decreased in prostate cancer cells. This suggests that PRH acts as a tumour suppressor protein in prostate cells. However, direct evidence to support this conclusion is lacking. Here we show that the *HHEX* gene that encodes PRH is often deleted in prostate cancer cells and that PRH mRNA levels and PRH protein levels are decreased in prostate cancer cells compared to normal cells. Using two different mouse models, we show that the expression of PRH in prostate cancer cells inhibits cell proliferation and reduces tumour formation as well as tumour growth. These findings demonstrate that the PRH protein functions as a tumour suppressor in prostate cells.

## 1. Introduction

Prostate cancer progression is driven by genetic and epigenetic changes that accumulate over time and in response to diverse triggers, including DNA replication errors, exposure to mutagens, and chronic inflammation. Prostate adenocarcinoma arises predominantly from the glandular epithelium, and high-grade prostatic intraepithelial neoplasia (PIN) is its earliest recognised precursor [1]. High-grade PIN frequently shares mutations with invasive carcinoma, including *TMPRSS2–ERG* gene rearrangements, mutations in *TP53*, and loss of the PTEN tumour suppressor gene at 10q23, confirming its role as a precursor to malignancy [2]. Tumour evolution involves the accumulation of mutations and large-scale chromosomal rearrangements such as those that occur during chromothripsis, the shattering and random re-ligation of a single chromosome or a small number of chromosomes [3,4,5]. In addition, prostate tumour cells usually display genome-wide hypermethylation that epigenetically represses the expression of genes required for cell differentiation as well as tumour suppressor genes [6,7].

The Proline Rich Homeodomain/Haematopoietically Expressed Homeobox (PRH/HHEX) transcription factor is encoded by the *HHEX* gene located at 10q23 [8]. The PRH protein is highly conserved in all complex animals, and it is required for organogenesis and vascular development in the embryo and for the regulation of cell proliferation and cell migration in multiple cell types in the adult (reviewed [9,10]). PRH forms highly stable homo-oligomeric complexes that bind to tandemly arrayed homeodomain binding motifs with high affinity and regulate gene transcription [11,12,13]. However, PRH can also regulate transcription via protein–protein interactions with other transcription factors, including GATA2 [14], SRF [15], and AP-1 [16], and PRH regulates the nuclear export of some mRNAs important in the cell cycle, including the Cyclin D1 mRNA, through binding to eIF4E [17].

PRH intracellular localisation is regulated by interaction with other proteins; for example, single-chain uPA reduces nuclear localisation of PRH in endothelial cells [18], and glypican-3 and CD81 regulate PRH localisation and activity in hepatic cells [19]. Additionally, PRH DNA binding activity and protein stability are regulated by Protein Kinase CK2-dependent phosphorylation of amino acids S163 and S167 within the PRH homeodomain [20]. Phosphorylation by CK2 prevents phosphorylated PRH (pPRH) from binding to DNA and, in chronic myeloid leukaemia cells, phosphorylation results in the processing of pPRH by the proteasome to form a pPRHΔC isoform that lacks the PRH C-terminal domain [20,21]. pPRHΔC acts as a dominant negative regulator of full-length PRH at least in part by the sequestration of corepressor proteins belonging to the TLE family that otherwise mediate PRH-dependent transcriptional repression [21].

Interestingly, increased or decreased PRH expression is associated with tumourigenesis (reviewed [22]). In bile duct epithelial cells that normally express little or no PRH, aberrant over-expression of PRH results in increased cell proliferation and increased cell migration [23]. Moreover, knockdown of PRH in PRH-expressing bile duct cancer cells inhibits cell proliferation and inhibits tumour initiation and tumour growth in a mouse xenograft model [23]. Similarly, in colorectal cancer cells, PRH expression is upregulated, and in this tumour type, pPRH is oncogenic through the promotion of YAP-TEAD4 interactions that stimulate cell proliferation [24]. While in hepatocellular carcinoma, PRH also plays an oncogenic role, acting with transcriptional co-activator ABI2 to drive increased cell proliferation and migration and increased cancer stem cell formation [25]. Moreover, in acute myeloid leukaemia, PRH represses Cdkn2a (p16INK4a/p19ARF) expression and thereby promotes myeloid leukaemogenesis [26].

In contrast, in other cell types, PRH appears to function as a tumour suppressor protein with loss of PRH function through decreased protein expression or mislocalization of PRH to the cytoplasm, associated with increased cell proliferation [22]. For example, cytoplasmic PRH protein localisation is increased in breast cancer cells, and PRH knockdown increased the proliferation of breast cancer cells [27,28]. Moreover, we showed that over-expression of PRH in breast cancer cells reduced tumour growth in a xenograft mouse model [28]. Similarly, in hepatocellular carcinoma cells, PRH interacts with the transcription factor MYC, which normally promotes cell proliferation, and, in these cells, PRH knockdown increased cell proliferation [29]. Furthermore, in skin cutaneous melanoma and trisomy 8 acute myeloid leukaemia, increased CpG methylation of the *HHEX* gene and decreased mRNA expression are associated with disease progression [30]. Taken together, these findings suggest that PRH is a member of a small group of proteins that act as proto-oncogenes with tumour suppressor function. That is, proteins that normally inhibit tumourigenesis or tumour progression, but which can promote tumourigenesis or tumour progression, when they are overexpressed or mutated.

Our previous work has shown that in prostate cancer cells, loss of PRH expression increased cell proliferation and cell migration [31]. Moreover, in a limited collection of clinical samples, pPRH protein levels appeared to be increased in benign prostatic hyperplasia (BPH) and to a lesser extent increased in prostate adenocarcinoma samples, compared to normal prostate epithelial cells [31]. These findings suggest that PRH is inactivated in BPH by increased phosphorylation and possibly by reduced protein expression. This is consistent with PRH playing a tumour-suppressive role in prostate cells; however, direct evidence to support this conclusion is limited. Here, we show that the *HHEX* gene is often deleted in prostate cancer cells and that PRH mRNA levels are reduced in prostate cancer cells compared to normal tissue. Moreover, over-expression of PRH in prostate cancer cells reduced cell proliferation and cell migration in vitro and inhibited tumour growth and tumour initiation in human and mouse prostate cancer xenograft models. We conclude that PRH does indeed function as a tumour suppressor protein in prostate epithelial cells.

## 2. Materials and Methods

### 2.1. Cell Culture

PC3 cells were originally isolated from a prostate adenocarcinoma bone metastasis [32] and were purchased from the ATCC(Manassas, VA, USA). DVL3 cells were isolated from prostate tumour tissue from a Pten^−/−^/trp53^−/−^ mouse [33] and were supplied by Professor Ian G. Mills (University of Oxford). HEK293 cells were purchased from the ATCC. PC3 cells and their derivatives were cultured in RPMI 1640 medium (Sigma-Aldrich, St. Louis, MO, USA) supplemented with 10% fetal bovine serum (FBS, F7524, Sigma-Aldrich), 2 mM L-glutamine, and 1% penicillin/streptomycin at 37 °C and 5% CO_2_ in a humidified chamber. DVL3 cells were cultured in RPMI 1640 medium with GlutaMAX and HEPES (Gibco, Inchinnan, Scotland) supplemented with 10% FBS (Gibco) and 100 nM 5α-Dihydrotestosterone (Merck, Darmstadt, Germany), as above. HEK293 cells were grown in Dulbecco’s modified Eagle medium (DMEM) (Fisher Scientific, Loughborough, UK) with 10% FBS, as above. All cell cultures were tested monthly for mycoplasma infection and kept for a maximum of 30 passages.

### 2.2. Virus Production and Cell Infection

A DNA sequence encoding amino acids 7-277 of human PRH and an N-terminal Myc tag were cloned into pcLVi(3G)-MCS-CopGFP-IRES-Neo (Sirion Biotech, Gräfelfing, Germany). A doxycycline-inducible Myc-tagged PRH expression lentivirus was then produced in HEK293 cells transiently transfected with pMDG2, psPAX2, and pcLVi(3G)-Myc-PRH-CopGFP-IRES-Neo using polyethyleneimine (PEI). DNA PEI complexes were assembled in Opti-MEM reduced serum medium (Fisher Scientific) following the supplier’s instructions. After 4 h at 37 °C and 5% CO_2_ in a humidified chamber, the medium was replaced, and the cells were grown for 48 h as above. The medium was then passed through a 0.4 μm pore filter, and the lentivirus particles were purified using a Lenti-X™ concentrator (Takara Bio, Kusatsu, Japan) following the manufacturer’s instructions. A polyclonal PC3 cell line with doxycycline-inducible Myc-PRH expression (PC3 PRH Lv) was then generated by lentiviral transduction and G418 selection. Three days after virus addition, G418 (3 mg/mL Fisher Scientific) was added to start the selection and the medium with G418 was changed every two days for three weeks before cell harvest and storage in liquid nitrogen. Myc-PRH expression was induced in thawed PC3 PRH Lv cells by the addition of doxycycline (2 μg/mL) to the media for 7 days, with fresh doxycycline and media added daily. Production and characterisation of an adenovirus vector expressing Myc-tagged PRH (Ad-PRH) has been described previously [34]. DLV3 cells were infected with Ad-PRH or Ad-empty virus at 100 MOI for 24 h at 37 °C and 5% CO_2_ in a humidified chamber as described above. The medium was then replaced with fresh medium, and the cells were left to recover for another 24 h before use.

### 2.3. Cell Viability and Cell Proliferation Assays

Cell viability was measured using an MTT assay (Merck M5655-1G) according to the manufacturer’s instructions and a VersaMax™ microplate reader (Molecular Devices, San Jose, CA, USA). Readings were normalised to vehicle control, and GraphPad Prism 11.0.2 was used to derive dose–response curves. Cell death was assayed using propidium iodide (Sigma-Aldrich) staining. Stained cells were imagined using a Leica DMI 6000 B inverted epifluorescence microscope (Leica Microsystems, Wetzlar, Germany) and analysed using ImageJ 1.54t to count propidium iodide-stained cells in a total of 10 fields/sample. Cell proliferation was measured using a Click-iT EdU Microplate Assay kit (Fisher Scientific), following the manufacturer’s instructions.

### 2.4. Cell Migration Assays

Cell migration assays were performed as described previously [35] using Boyden chambers (Greiner Bio-One, Kremsmünster, Austria) with RPMI 1640 medium and 2% FBS in the chamber and 10% FBS in the well. Calcein-AM (Fisher Scientific) was used to stain the migrated and non-migrated cells, and staining was measured using black flat-bottom 96-well plates and a FlexStation microplate reader (Molecular Devices, San Jose, CA, USA).

### 2.5. Western Blotting

Cell extracts were prepared by sonication in cell lysis buffer (Cell Signalling Technology, Danvers, AM, USA) with 1 uM PMSF and PhoStop (Sigma-Aldrich). Proteins were separated by SDS-PAGE and transferred to a PVDF membrane using a wet transfer system (BioRad, Watford, UK). Western blotting was then performed using the following primary antibodies: β-actin 4970S (Cell Signalling Technology), PRH/HHEX 2018B FAB83771C (BioTechne, Minneapolis, MN, USA), cleaved caspase 3 MAB835 (BioTechne), and Myc 9E10 MA1-980 (Fisher Scientific).

### 2.6. qRT-PCR

Total RNA was extracted using a Bioline Isolate II kit (Bioline, London UK) and reverse transcribed using a Quantitect Reverse Transcription kit (Qiagen 205311, Hilden, Germany) according to the manufacturer’s instructions. RT-qPCR was performed using a Rotor-Gene Q cycler (Qiagen) with a Quantitect SYBR green PCR kit (Qiagen, 204143). The expression of genes of interest was normalised to Glyceraldehyde-3-phosphate dehydrogenase (GAPDH) or β-actin mRNA using the Pfaffl method [36]. The primers used are shown in Table 1.

### 2.7. Chromatin Immunoprecipitation

PC3 cells were infected with Ad-PRH or Ad-empty as described above, and the cells were harvested using a non-enzymatic cell dissociation solution (Fisher Scientific). Chromatin immunoprecipitation was then performed as previously described [11,21], and DNA fragments were isolated using an anti-Myc-tag antibody or rabbit IgG antibody attached to Protein G bead (Dynabeads, Fisher Scientific). Quantitative PCR was performed as above in 4 replicates, with DNA from 3 independent experiments. Primers were designed based on the PRH ChIP peaks identified in CCLP cells, and sequences from chromosome 18 were used as a negative control. ChIP primers are shown in Table 2.

### 2.8. RNA Sequencing

RNA was isolated using a Bioline Isolate II kit as above, and poly-adenylated RNA was then purified using a TruSeq RNA Library Prep kit (Illumina, San Diego, CA, USA) according to the manufacturer’s instructions. Illumina sequencing of 75 bp paired-end reads (minimum 2 × 35 million reads/sample) was performed in biological duplicate. Bioinformatic analysis was performed on the Galaxy platform [37]. Raw sequencing reads were quality-assessed with FastQC, followed by adapter removal and quality trimming using TrimGalore. Cleaned reads were aligned to the reference genome (hg19) using TopHat. Read counts were then generated with featureCounts, and differential expression analysis was conducted using DESeq2. Subsequent downstream analyses, including gene set enrichment analysis (GSEA) and the generation of volcano plots, were performed outside of Galaxy using GSEA 4.3.x software [38] and VolcaNoseR 1.0.3 [39], respectively.

### 2.9. TMA Staining

A commercial prostate cancer tissue array (ab178264 Abcam, Cambridge, UK) with 96 samples from 48 cases demonstrating different degrees of neoplasia, ranging from BPH to advanced prostate adenocarcinoma, was stained for PRH using an in-house monoclonal PRH antibody (PRH M6) that has been described previously [31,35]. The images were scored following Gleason’s scoring system with modifications made by the 2005 International Society of Urological Pathology (ISUP) Consensus Conference on Gleason Grading of Prostatic Carcinoma. PRH staining was scored using the H-Score method [40]. The intensity (no staining 0, weak staining 1, moderate 2, and strong 3) and frequency of stained cells (from 0% to 100%) were multiplied to obtain a PRH staining score. The latter is a range between 0 and 300. This method was used to score nuclear and cytoplasmic PRH staining. Before scoring, the first investigator underwent training for histological assessment and recognition of various morphological categories (normal tissue, hyperplasia, malignancy) and immunohistochemical analysis of PRH protein expression, including nuclear and cytoplasmic expression assessment. All cases were assessed in duplicate, and duplicate cores from the same patient were then averaged to provide a final score. Cases were analysed by the first investigator, then reviewed by a senior pathologist. Scores were overall concordant, with only minor revision of the scores by the pathologist where non-lesional tissue was included.

### 2.10. Mouse Xenograft Models

PC3 PRH Lv cells were grown as described above before subcutaneous injection into CD1 male nude mice (5 × 10^6^ cells in 100 µL per mouse). Tumour growth was then monitored three times per week using callipers, and once tumours reached 3 mm × 3 mm, the animals were divided into 2 groups (n = 6 per group) to receive either normal drinking water or doxycycline-supplemented water (2 mg/mL doxycycline in 5% sucrose). Tumour growth was then monitored three times per week as above, and tumour volumes calculated using the formula: [(length + width)/2] ∗ length ∗ width. Statistical analysis was performed using a two-way ANOVA (GraphPad).

To assess tumour initiation, PC3 PRH Lv cells were grown as described above and treated daily with either DMSO vehicle or doxycycline (2 μg/mL) for 13 days with the daily addition of fresh doxycycline before subcutaneous injection into CD1 male nude mice (5 × 10^6^ cells in 100 µL per mouse, n = 6 per group). Once the tumours reached 3 mm × 3 mm, tumour growth was monitored three times per week as above. PC3 PRH Lv-vehicle tumours reached 3 mm × 3 mm by day 13 post-injection, and quantification began on day 13 as day 1. No tumours developed in the PC3 PRH Lv-doxycycline group for 42 days. Tumour volumes were measured and analysed as above.

DVL3 cells were infected with Ad-empty or Ad-PRH at 100 MOI, and after 24 h, 10^6^ cells in 100 µL were subcutaneously injected into C57BL mice. Tumour volume was measured as above over 3 weeks or until the tumours reached the maximum allowed diameter (12 mm) when the mice were culled.

To examine the effects of CX4945 treatment on tumour growth, DVL3 cells were subcutaneously injected into C57BL mice (10^6^ cells in 100 µL) and tumour growth was measured and analysed as described above. Once the tumours reached 3 mm × 3 mm, the animals were divided into 2 groups (n = 6 per group) to receive either 25 mM NaH_2_PO_4_ buffer (5% DMSO) as a control or 20 mg/kg CX4945 in 25 mM NaH_2_PO_4_ buffer (5% DMSO) intraperitoneally three times per week. Tumour volumes were measured and analysed as above.

### 2.11. Statistical Analysis

Experiments were repeated to a minimum of N = 3 biological repeats, and statistical analyses were performed using GraphPad Prism 10.0 (GraphPad Software, San Diego, CA, USA). Error bars represent standard error of the mean (SEM), unless stated otherwise and a *p*-value lower than 0.05 was accepted as statistically significant. For the numerical variables with a normal distribution, a two-tailed unpaired *t*-test was performed. For the numerical variables with multiple group comparisons, a standard two-way ANOVA followed by a Bonferroni post-test was used. Correlation tests were made with Pearson’s correlation test, assuming that the data present a normal distribution. Pearson’s r values between 0 and ±0.3 were considered weak, values between ±0.3 and ±0.7 were considered moderate, and values between ±0.7 and ±0.99 were considered strong. The r2 values were used to express how much of the variability in the first variable was explained by the second variable in percentage.

## 3. Results

### 3.1. The HHEX Gene Encoding PRH Is Altered in Prostate Adenocarcinoma Cells

**Figure 1:**
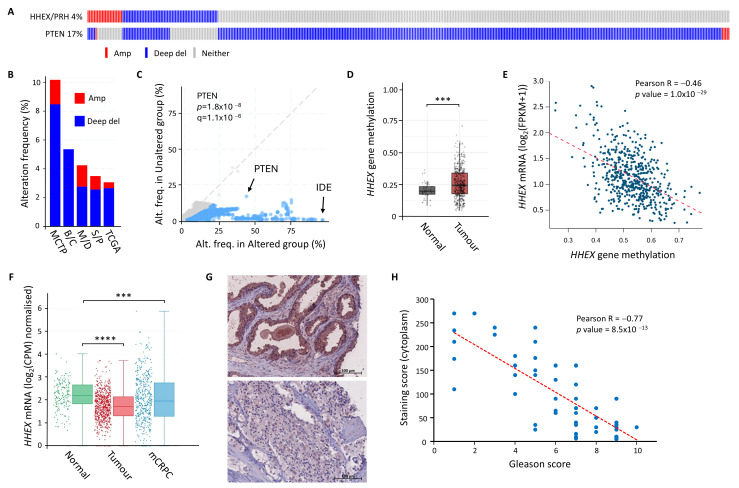
PRH expression is decreased in prostate cancer cells by multiple mechanisms. (**A**) Data from five studies with a total of 1976 patients were analysed for *HHEX* and *PTEN* gene amplification and deep deletion events using cBioPortal. Each bar represents a patient. (**B**) A graph showing the individual results from each study in (**A**): MCTP-Metastatic Prostate Adenocarcinoma [46], B/C-Prostate Adenocarcinoma [47], M/D-Prostate Adenocarcinoma [48], S/P-Metastatic Prostate Adenocarcinoma [49], TCGA-Prostate Adenocarcinoma [50]. (**C**) Analysis of genes co-deleted with *HHEX* in the studies from (**B**). (**D**–**F**) PRH mRNA and *HHEX* gene CpG methylation data produced using TCGA data and SMART [51]: (**D**) *HHEX* gene CpG methylation levels in prostate adenocarcinoma and normal prostate samples. Mean and SEM. (**E**) A scatter plot of *HHEX* mRNA expression (log_2_(FPKM + 1)) and *HHEX* gene CpG methylation levels (β value). (**F**) *HHEX* mRNA expression in normal prostate samples, primary prostate adenocarcinoma (tumour) and metastatic castration-resistant prostate cancers (mCRPC) was produced using the Prostate Cancer Atlas [52]. *** = *p* < 0.005, **** = *p* < 0.0005. (**G**) Images from a TMA stained with a PRH monoclonal antibody as described in the text. Top—a representative example of a sample with a good number of well-differentiated glands and moderately strong and highly frequent PRH cytoplasmic staining. Bottom—a representative sample with no PRH cytoplasmic staining. (**H**) A scatter plot of cytoplasmic PRH immunostaining score versus Gleason score showing moderate to strong negative correlation.

To examine the relevance of PRH to prostate adenocarcinoma, cBioportal [41] was used to analyse gene alterations in prostate cancer cases. Data from five different studies were analysed, giving a total of 1976 patients (Figure 1A,B). The *HHEX* gene encoding PRH showed shallow heterozygous gene deletion in 25.9% of these samples, while deep homozygous deletions and gene amplifications were present in 3.2% and 1.0% of the samples, respectively (Figure 1A). The difference in the frequency of deletions observed in the different studies is likely due to the different number of samples in each case, and in the largest study, the frequency of deep gene deletions was 2.8%. In the same group of patients, the *PTEN* tumour suppressor gene showed deep deletion in 17% of the cases (Figure 1A). Interestingly, co-deletion of *HHEX* and *PTEN* occurred in 31 cases, and co-occurrence was statistically significant (*p* = 1.8 × 10^−8^, q = 1.1 × 10^−6^). To examine this in more detail, we looked at the genes co-deleted with *HHEX* more generally (Figure 1C). *KIF11* and *IDE* were the most frequently co-deleted genes (73% co-deleted in each case). These genes lie immediately upstream of the *HHEX* gene at chromosome 10q23. The *PTEN* gene lies approximately five megabases further upstream, and it was co-deleted in 37% of *HHEX*-deleted cases. In conclusion, the *HHEX* gene encoding PRH is frequently deleted in prostate adenocarcinoma, and this can occur with or without co-deletion of *PTEN*.

Gene expression can be silenced by CpG methylation, and this is important in the down-regulation of several tumour suppressor genes in different cancer types, including the *RB1* gene in retinoblastoma [42] and *CDKN2A (p16)* gene in bladder, breast, prostate, renal, and colon cancers [43,44]. Interestingly, CpG methylation of the *HHEX* gene was statistically significantly increased in prostate adenocarcinoma samples compared to normal tissue (Figure 1D and Figure S1). Moreover, there was a correlation between increased CpG methylation in the *HHEX* gene and decreased PRH mRNA expression in prostate samples (Figure 1E), with a moderate correlation (R = −0.46) between the two variables. This is consistent with the decreased PRH mRNA levels seen in prostate adenocarcinoma and castration-resistant prostate cancer compared to normal samples (Figure 1F). Thus, there is increased CpG methylation of the *HHEX* gene in the prostate cancer samples, and this correlates with down-regulation of PRH mRNA expression.

### 3.2. PRH Protein Expression Is Lower in More Advanced Prostate Adenocarcinomas

In a previous study, we examined PRH and pPRH expression in a small number of prostate tissue samples [31]. This study demonstrated that pPRH was elevated in BPH and prostate adenocarcinoma compared to normal prostate epithelial cells, while total PRH staining appeared to be similar in BPH, prostate adenocarcinoma and normal prostate epithelial cells [31]. However, this study did not examine sufficient prostate adenocarcinoma samples to determine whether PRH protein levels are altered in advanced prostate adenocarcinoma. To examine PRH protein expression in a larger number of samples, we made use of a tissue microarray (TMA) with 96 prostate hyperplasia and adenocarcinoma samples obtained by needle biopsy. The TMA was stained for PRH expression, and the samples were scored using the Gleason scoring system [45], as described in Section 2.

A representative image of a low Gleason score sample with a number of well-differentiated glands is shown in Figure 1G (top panel). PRH staining in this sample was moderately strong and highly frequent in the cytoplasm and nucleus of the epithelial cells. In contrast, Figure 1G (bottom panel) shows a representative image of a high Gleason score sample. In this sample, the largest area showed infiltration of glandular structures (grade 3), while a second, bigger, distinctive area showed irregular solid nests of tumour cells (grade 4). In this case, PRH staining was absent in the cytoplasm and nucleus. The results from the analyses were compiled in a scatter plot, and Pearson’s correlation test showed a moderate to strong negative correlation between the Gleason score and PRH cytoplasmic staining intensity (Figure 1H) (R = −0.77, *p* = 8.5 × 10^−13^). Around 60% of the variance in cytoplasm PRH staining was explained by the Gleason score. A similar trend was observed between the Gleason score and nuclear PRH staining, but with a weak to moderate negative correlation (R = −0.39, *p* = 2.4 × 10^−3^) (Figure S2A) and there was a moderate positive correlation between the cytoplasmic and nuclear staining score (R = 0.47, *p* = 1.9 × 10^−4^) (Figure S2B). Together, these results show that PRH was highly expressed in the well-differentiated tumours and weakly expressed in the less-differentiated tumours.

### 3.3. PRH Over-Expression in PC3 Cells Inhibits Cell Proliferation and Cell Migration

**Figure 2:**
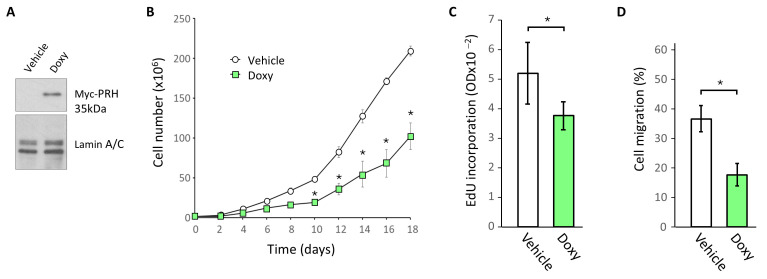
PRH over-expression in PC3 prostate cancer cells inhibits cell proliferation and cell migration. (**A**) PC3 PRH Lv cells were treated with vehicle or doxycycline (0.3 ug/mL) for seven days before whole cell extracts were prepared for Western blotting. A Myc-tag antibody was used to detect Myc-PRH, and a Lamin A/C antibody was used to detect Lamin A/C as a loading control. (**B**) PC3 PRH Lv cells were treated with vehicle or 1.5 ug/mL doxycycline, and cell number was counted over eighteen days. Mean and SEM. Two-way ANOVA * = *p* < 0.05. When error bars cannot be seen, they are smaller than the symbols. (**C**) PC3 PRH Lv cells were treated with 1.5 ug/mL doxycycline or vehicle for seven days, and EdU incorporation was then measured using a colorimetric assay. Mean and SEM of 3 biological repeats. Two-tailed unpaired *t*-test, * = *p* < 0.05. (**D**) PC3 PRH Lv cells were treated with vehicle or doxycycline for 7 days as in (**C**), and cell migration was then assessed using a Transwell chemotaxis assay. Mean and SEM of 3 biological repeats. Two-tailed unpaired *t*-test, * = *p* < 0.05. Original Western blots are shown in Figure S6.

To inducibly over-express PRH in a prostate cancer cell line, PC3 cells were transduced using lentivirus particles carrying a Myc-tagged PRH cDNA downstream of a doxycycline-inducible promoter. The lentivirus conferred gentamicin resistance, and G418 was used to select transduced cells. To confirm that PRH over-expression was induced by doxycycline treatment, the resulting polyclonal PC3 PRH Lv cell line was treated with vehicle or doxycycline (0.3 μg/μL) for seven days, and Western blotting was then used to examine Myc-PRH expression (Figure 2A). As expected, doxycycline treatment resulted in the expression of Myc-PRH in the treated cells. To examine the effects of PRH over-expression on PC3 PRH Lv cells, the cells were treated with doxycycline (1.5 μg/μL) over eighteen days, and cell number was determined every two days by counting (Figure 2B). Treatment with this concentration of doxycycline reduced the number of PC3 PRH Lv cells over time (Figure 2B). Importantly, this concentration of doxycycline had little or no effect on the viability of PC3 cells as measured using an MTT assay (Figure S3). To examine whether the decrease in cell number was accompanied by a reduction in cell division, an EdU incorporation assay was used to determine the number of cells transitioning through S-phase (Figure 2C). EdU incorporation was significantly reduced in the doxycycline-treated PC3 PRH Lv cells when compared to vehicle treatment (*p* < 0.05). In contrast, treatment of PC3 PRH Lv cells with this concentration of doxycycline did not increase the number of dead cells (Figure S4). Thus, the decrease in the number of doxycycline-treated PC3 PRH Lv cells is likely due in large part to reduced cell division.

In breast and prostate epithelial cells, PRH over-expression inhibited cell migration and invasion through the transcriptional up-regulation of the TGFβ co-receptor Endoglin [35] and, in a non-small cell lung cancer model, PRH was shown to inhibit cell migration through decreased RHOA/CDC42-dependent signalling [53]. To examine whether PRH over-expression in this model represses cell migration, PC3 PRH Lv cells were treated with vehicle or doxycycline (1.5 ug/mL) for 7 days before being tested in a chemotaxis assay over 24 h and in the presence of hydroxyurea, which inhibits cell proliferation. Doxycycline treatment under these conditions resulted in a ~50% reduction in the number of migrated cells (Figure 2D).

### 3.4. PRH Over-Expression Regulates Genes Associated with Cell Proliferation and Migration

**Figure 3:**
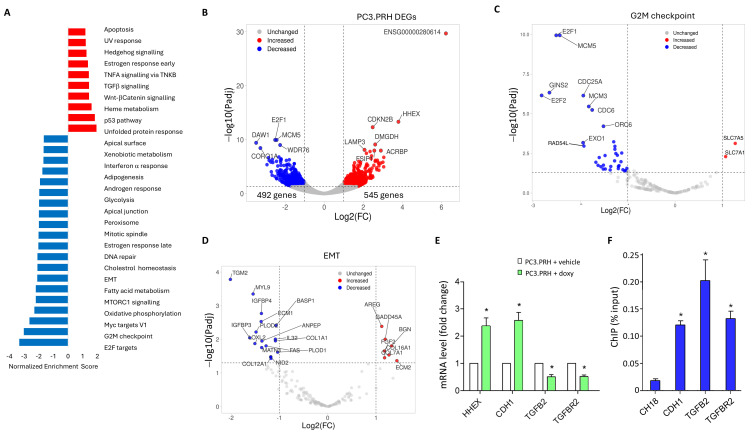
PRH over-expression alters the expression of genes involved in cell proliferation and epithelial–mesenchymal transition. (**A**) PRH over-expression was induced in PC3 PRH Lv cells, and mRNA expression was determined using RNA seq as described in the text. Pre-ranked GSEA using the Wald statistic identified significantly altered Hallmark gene sets. (**B**) The volcano plot shows the differentially expressed genes following doxycycline treatment of PC3 PRH Lv cells. *HHEX* mRNA up-regulation indicates successful PRH mRNA induction. (**C**) G2M checkpoint- and (**D**) EMT-related differentially expressed genes following doxycycline treatment of PC3 PRH Lv cells. (**E**) RT-qPCR analysis of selected genes in PC3 PRH Lv cells treated with vehicle or 1.5 ug/mL of doxycycline. Statistical comparisons made were between doxycycline-treated and vehicle: one-sample *t*-test with hypothetical value set to ‘1’, * = *p* < 0.05, N = 3. (**F**) Quantitative PCR using chromatin immunoprecipitated by Myc-tagged PRH or control IgG. Percentage input: one-way ANOVA, * = *p* < 0.05, N = 3.

To identify genes that are differentially expressed following the induction of PRH expression, we isolated mRNA from PC3 PRH Lv cells and PC3 cells that had each been treated with either vehicle alone or doxycycline. Treatment of PC3 PRH Lv cells with doxycycline resulted in 1037 differentially expressed genes (adjusted *p* < 0.05, |log_2_FC| ≥ 1), while treatment of PC3 cells with doxycycline resulted in only 54 differentially expressed genes. Gene set enrichment analysis (GSEA) was performed using a pre-ranked approach, with genes ranked according to the Wald statistic obtained from DESeq2 differential expression analysis. Enrichment analysis was conducted using the Hallmark gene set collection (MSigDB H). A total of 1000 permutations were applied, and gene sets with a false discovery rate (FDR) q-value < 0.25 were considered significantly enriched. Genes associated with the control of cell proliferation, including G2M checkpoint genes, Myc target genes, and E2F target genes, were significantly negatively enriched with PRH over-expression (Figure 3A). GSEA also showed negative enrichment of genes associated with EMT and cell migration (Figure 3B). Analysis of the full data set showed that, as expected, the *HHEX* gene encoding PRH was overexpressed in the set of 545 significantly upregulated genes, and increased *HHEX* mRNA expression was confirmed using RT-qPCR (Figure 3E). In marked contrast, *E2F1* and *MCM5*, which are both important for cell cycle progression, were significantly repressed genes in the 492 down-regulated genes. Analysis of G2M checkpoint-associated genes showed that many of these genes were repressed in the presence of over-expressed PRH (Figure 3C), and this is consistent with the down-regulation of cell proliferation by PRH seen in Figure 2. Moreover, PRH over-expression resulted in down-regulation of the genes encoding TGFβ and TGF receptor II (Figure 3E) and TGFβ signalling is known to increase the proliferation of prostate cancer cells [54]. Overexpressed Myc-tagged PRH was seen to bind both of these genes in chromatin immunoprecipitation experiments (Figure 3F).

Analysis of EMT-associated genes showed that while some genes were increased in expression in the presence of overexpressed PRH, many more genes were repressed, including *TGM2* (Figure 3D), which encodes Transglutaminase 2, the activity of which promotes castration-resistance and represses transcription of the androgen receptor in prostate cancer [55]. A role for PRH in the inhibition of prostate cell migration and the inhibition of epithelial to mesenchymal transition (EMT) is further supported by the up-regulation of *CDH1*, which encodes E-cadherin (Figure 3E), an epithelial cell marker and well-characterised inhibitor of cell migration and EMT [56]. Chromatin immunoprecipitation showed that PRH binds to the *CDH1* gene (Figure 3F and Figure S7). This is consistent with our previous studies, which showed that over-expression of PRH in PC3 cells increased expression of E-Cadherin while PRH knockdown in normal immortalised PNT2-C2 cells resulted in decreased E-Cadherin expression [31,54]. However, it is likely that many of these DEGs contribute to the inhibition of cell migration seen in the presence of PRH over-expression.

### 3.5. PRH Over-Expression Inhibits Tumour Growth and Tumour Initiation In Vivo

**Figure 4:**
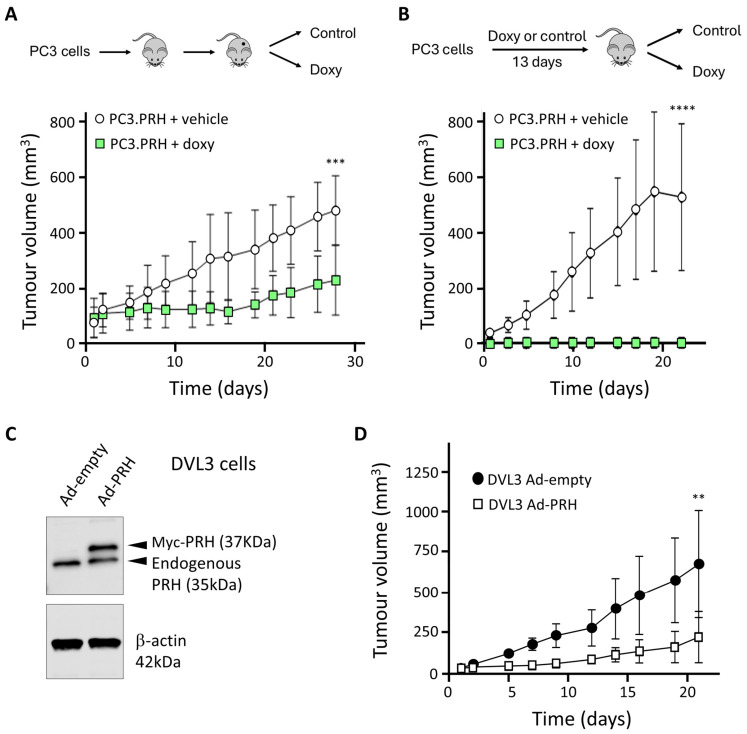
PRH over-expression represses tumour growth and tumour initiation. (**A**) PC3 PRH Lv cells were subcutaneously injected into CD1 male nude mice, and when the tumours reached 3 mm × 3 mm, the mice were divided into 2 groups (n = 6 per group) to receive normal drinking water or drinking water containing doxycycline (2 μg/mL) in 5% sucrose (filled symbols). Mean and SEM. Two-way ANOVA (*** = *p* < 0.0005). (**B**) PC3 PRH Lv cells were grown in the presence of DMSO vehicle (open symbols) or doxycycline (2 μg/mL) (filled symbols) for 13 days before subcutaneous injection into CD1 male nude mice (n = 6 per group). Mean and SEM. Two-way ANOVA (**** = *p* < 0.0001). When error bars cannot be seen, they are smaller than the symbols. (**C**) DVL3 cells were infected with Ad-empty or Ad-PRH at an MOI of 100 for 48 h, and whole cell extracts were then prepared for Western blotting. PRH and Myc-tagged PRH were detected using a PRH monoclonal antibody, and β-actin was used as a loading control. (**D**) DVL3 cells were infected with Ad-empty or Ad-PRH as above and then subcutaneously injected into C57BL mice (n = 3 per group). Mean and SEM. Two-way ANOVA (** = *p* < 0.005). Original Western blots are shown in Figure S6.

The data described above suggest that the loss of PRH activity in prostate cancer cells may contribute to tumour growth. To test this hypothesis, we subcutaneously injected PC3 PRH Lv cells into immunodeficient CD1 male nude mice and allowed the resulting tumours to grow until they were 3 mm × 3 mm in size. The mice were then divided into two groups (n = 6) to receive either normal drinking water or doxycycline-supplemented drinking water, and tumour growth was monitored three times per week over four weeks. As expected, in the mice receiving normal drinking water, the tumours increased in size at a constant rate over the four weeks of the experiment (Figure 4A). However, in the mice receiving doxycycline, little or no tumour growth was observed until day 18 of treatment, and by the end of the experiment, the average tumour volume was less than half of that seen in the control group (*p* ≤ 0.0005). We conclude that PRH over-expression inhibited tumour growth in this model.

To determine whether PRH over-expression in PC3 cells also inhibits tumour-initiating potential, PC3 PRH Lv cells were grown in the presence of either DMSO vehicle or doxycycline for 13 days before subcutaneous injection into CD1 male mice. Mice injected with PC3 PRH Lv-vehicle-treated cells developed tumours reaching 3 mm × 3 mm by day 13 post-injection (which is day 1 in Figure 4B), and the tumours continued to grow over the course of a further 24 days (Figure 4B). In marked contrast, no tumours developed in mice receiving the PC3 PRH Lv cells grown in the presence of doxycycline over 42 days (Figure 4B ). These data suggest that PRH over-expression in PC3 cells results in impaired tumour-initiating potential and supports a tumour-suppressive role for PRH.

To examine whether the method of PRH over-expression or the mouse model used in these experiments could be responsible for the inhibitory effects of PRH on tumour growth, we made use of an alternative expression method and an alternative mouse model. We have shown previously that the delivery of a Myc-tagged PRH construct to prostate cancer cells using an Adenoviral vector results in the inhibition of cell proliferation and the inhibition of cell migration [31]. To determine whether over-expression of PRH using this method inhibits the growth of prostate cancer cells as tumours, we made use of murine DVL3 prostate cancer cells. DVL3 cells lack PTEN and p53 expression, but, unlike PC3 cells, they are androgen-sensitive. Moreover, DVL3 cells form tumours in immunocompetent mice. DVL3 cells were infected with the Ad-PRH construct or Ad-empty, and the expression of Myc-PRH in the Ad-PRH-infected cells was confirmed by Western blotting (Figure 4C). Under the conditions used in this experiment, a modest level of PRH over-expression was achieved in the Ad-PRH-infected DVL3 cells. To examine the effects of PRH over-expression on the growth of DVL3 cells, DVL3 cells infected with Ad-empty or Ad-PRH were subcutaneously injected into immunocompetent C57BL mice and tumour growth was measured over 3 weeks. DVL3 cells infected with the control Ad-empty virus produced rapidly growing tumours in the injected mice (Figure 4D). However, DVL3 cells infected with Ad-PRH formed slow-growing tumours (Figure 4D). Thus, PRH over-expression using Ad-PRH resulted in a major reduction in the growth of murine DVL3 tumours in immunocompetent mice.

### 3.6. Inhibition of Protein Kinase CK2 Increases PRH Levels and Decreases Tumour Growth

**Figure 5:**
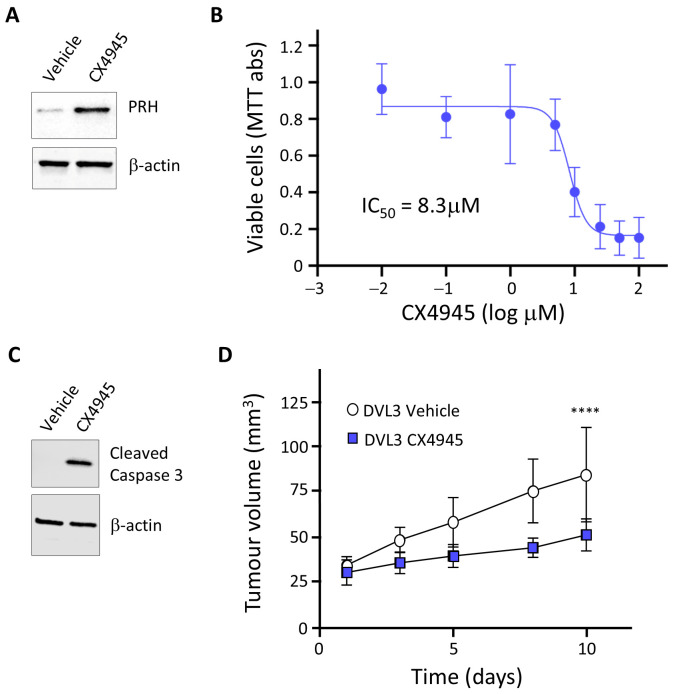
Inhibition of Protein Kinase CK2 increases PRH levels in DVL3 cells and decreases tumour growth. (**A**) DVL3 cells were treated with vehicle control or CX4945 (25 μM) for 48 h, and whole cell extracts were then prepared for Western blotting. PRH was detected using a PRH monoclonal antibody, and β-actin was detected as a loading control. (**B**) DVL3 cells were treated with increasing concentrations of CX4945 for 48 h, and cell viability was then measured using an MTT assay. Mean and SEM. N = 5 biological repeats. (**C**) DVL3 cells were treated with vehicle control or CX4945 as in (**A**), and Western blotting was then performed using a cleaved caspase 3 antibody and a β-actin antibody as a loading control. (**D**) DVL3 cells were subcutaneously injected into C57BL mice, and when the tumours reached 3 mm × 3 mm, the mice were divided into 2 groups (n = 6 per group) to receive 20 mg/kg CX4945 or vehicle control by intraperitoneal injection three times per week. Tumour volume measured and analysed as in Figure 4A. Mean and SEM. Two-way ANOVA (**** = *p* < 0.0001). Original Western blots are shown in Figure S6.

The data described above suggest that treatments which increase PRH mRNA expression levels, or PRH protein levels, in prostate cancer cells could decrease tumour growth. Our previous work showed that the phosphorylation of PRH by Protein Kinase CK2 results in decreased PRH protein levels and decreased PRH activity [20,21]. To determine whether the inhibition of CK2 activity results in an increase in PRH protein levels in prostate cancer cells, we treated DVL3 cells with the well-characterised CK2 inhibitor CX4945 [57]. Treatment of DVL3 cells with CX4945 resulted in increased PRH protein levels compared to vehicle-treated cells (Figure 5A). To examine the effects of CK2 inhibition on DVL3 cells, we performed MTT assays in the presence of increasing amounts of CX4945. Treatment with CX4945 resulted in a dose-dependent decrease in the number of viable cells (Figure 5B). Similar results were obtained in PC3 cells (Figure S5). Thus, the increase in PRH protein expression correlates with decreased tumour cell viability. The decrease in DVL3 cell viability was accompanied by increased levels of the apoptosis effector cleaved Caspase 3 (Figure 5C), suggesting that CX4945 treatment induced apoptosis in these cells. Moreover, CX4945 treatment reduced the growth of DVL3 cells as tumour xenografts in immunocompetent mice (Figure 5D). These data indicate that the restoration of PRH expression in prostate cancer cells is achievable using drug treatments that inhibit CK2, and they show that the inhibition of CK2 activity resulted in a decrease in tumour growth.

## 4. Discussion

While it has been suggested that PRH plays a tumour-suppressive role in prostate epithelial cells [31], direct evidence to support this is lacking. Here, we examined PRH mRNA expression and genetic/epigenetic alteration across large prostate cancer patient cohorts. The *HHEX* gene encoding PRH was homozygously deleted in 3.2% of the sequenced prostate adenocarcinoma samples, and heterozygously deleted in around 26% of the samples. Moreover, increased CpG methylation of the *HHEX* gene was observed in advanced prostate cancer, and this correlated with decreased PRH mRNA expression. This is in agreement with previous studies, which reported increased *HHEX* gene methylation and decreased mRNA expression in cutaneous melanoma and trisomy 8 AML [29,30]. It is likely that in the prostate samples with heterozygous deletion of the *HHEX* gene, the second allele may be inactivated by increased CpG methylation.

In our previous study with a more limited collection of clinical samples, of nine adenocarcinoma samples examined, four were graded GS > 9 and presented moderate PRH staining intensity, while three out of five tumours graded GS = 9 presented low intensity staining [31]. This is in agreement with the results presented here, which showed that PRH expression in the cytoplasm, and to a lesser extent in the nucleus, of prostate cancer samples is negatively correlated to the degree of differentiation of the tumours, expressed in the form of a Gleason score. During breast cancer progression, PRH activity is reduced in part at least by changes in its intracellular localisation, with increased PRH cytoplasmic localisation seen in advanced cancer [27,28]. However, we observed decreased cytoplasmic PRH staining in advanced prostate cancer samples. This suggests that although PRH mRNA levels are reduced in both breast and prostate cancer cells, and CpG methylation of the *HHEX* gene is increased in both cancer types, increased cytoplasmic localisation may be a feature of breast cancer cells but not prostate cancer cells.

Since PRH protein and PRH mRNA levels were decreased in advanced prostate cancer cells, we examined the effects of PRH over-expression on PC3 prostate cancer cells using a doxycycline-inducible cell line. The induction of PRH expression in these cells resulted in the inhibition of cell proliferation and the down-regulation of genes that promote cell proliferation, including E2F1 and E2F2. Other PRH-repressed genes include *TGM2*, which promoted the growth of W480 colorectal cancer cells as tumour xenografts in immunodeficient mice [58]. Moreover, PRH over-expression down-regulated multiple genes involved in TGFβ signalling and Myc-tagged PRH was shown to bind to these genes [54]. Down-regulation of these genes by PRH would also be expected to inhibit cell proliferation and cell migration. Interestingly, TGFβ down-regulates PRH protein and mRNA levels in prostate epithelial cells, suggesting that TGFβ signalling and PRH activity are in a regulatory loop. The induction of PRH over-expression in PC3 cells also resulted in reduced cell migration. Lower migratory ability is a characteristic of cells displaying an epithelial phenotype, and RNA sequencing showed that PRH over-expression in these cells resulted in the regulation of multiple genes associated with epithelial–mesenchymal transition. PRH increased E-cadherin mRNA levels, and Myc-tagged PRH was shown to bind to the *CDH1* gene in chromatin immunoprecipitation experiments. This is in agreement with our previous study, which showed that the expression of Myc-tagged PRH in these cells increased E-cadherin protein levels [54].

The induction of PRH expression with doxycycline in androgen-insensitive, PTEN-negative PC3 cells significantly slowed tumour growth in a xenograft mouse model. Moreover, pre-induction of PRH over-expression in PC3 cells before injection completely prevented tumour formation, demonstrating that tumour initiation was inhibited. The inhibitory effects on tumour growth were confirmed using an alternative PRH delivery method and an alternative mouse model. Adenoviral over-expression of PRH in murine androgen-sensitive, PTEN-negative DVL3 prostate cancer cells significantly reduced tumour growth in immunocompetent mice. These results are supported by our previous studies, which showed that PRH knockdown in immortalised but non-tumourigenic PNT2-C2 prostate epithelial cells resulted in increased cell migration and invasion, increased colony formation in Matrigel, and increased numbers of CD44+/CD133+ cells indicative of the self-renewing stem cell population [31]. Taken together, these findings indicate that PRH does indeed act as a tumour suppressor protein in prostate cells, most likely because its normal function in these cells is to down-regulate cell proliferation. Moreover, tumour suppression does not depend on the genetic background of the mouse cells or their immunocompetence.

Deletion of the *HHEX* gene encoding PRH often occurs with co-deletion of the *PTEN* tumour suppressor gene. *PTEN* and *HHEX* are both located on chromosome 10 in the region 10q23, and this region shows loss of heterozygosity in approximately 50% of prostate cancers [4]. Co-deletion of *HHEX* and *PTEN* does not occur in all cases with a deep deletion, suggesting that they act independently as tumour suppressor genes in prostate cancer. However, their frequent co-deletion could indicate that *HHEX* gene loss acts in concert with *PTEN* gene loss either additively or synergistically. Moreover, these findings confirm that the 10q23 region contains multiple genes that contribute to prostate tumour progression. This may be analogous to the cluster of genes that includes the *TP53* tumour suppressor gene, and other genes thought to act as tumour suppressor genes on chromosome 17p13.1, where there is evidence that wide 17p deletions are more damaging than the loss of the *TP53* gene alone [59]. Thus, 10q23 deletions may also have different phenotypes depending on the genes that are lost.

Since PRH protein expression is decreased in prostate cancer cells, the restoration of PRH expression may be a useful strategy to reduce tumour growth and tumour metastasis. Our previous studies showed that the regulatory subunit of Protein Kinase CK2 binds to the N-terminal domain of PRH and the catalytic subunit of CK2 phosphorylates the PRH homeodomain, resulting in the inhibition of DNA binding activity and increased cleavage of PRH by the proteasome [21,60]. Interestingly, PRH is hyper-phosphorylated in BPH, prostatic adenocarcinoma and prostate cancer cell lines, and PRH phosphorylation in prostate cells is dependent on CK2 activity [31]. Moreover, PRH phosphorylation by CK2 reduced PRH activity and increased prostate cancer cell migration and invasion [31].

Here, we have shown that an inhibitor of Protein Kinase CK2 increased PRH protein levels in mouse prostate cancer cells and decreased cell viability. The same drug inhibited the growth of these cells as xenograft tumours in mice, and others have shown that the inhibition of CK2 reduced the growth of PC3 cells as tumours in a mouse model [61]. Protein Kinase CK2 has multiple targets within these cells, and reducing the phosphorylation of many of these targets likely contributes to the effects of CK2 inhibition on tumour growth. Consistent with this view we have not been able to demonstrate that the removal of the PRH protein from prostate cancer cells abrogates the negative effects of CK2 inhibition on the proliferation of these cells. However, we have shown that PRH knockdown in non-tumourigenic PNT2-C2 cells reduces the effect of CK2 inhibition on these cells [31]. This suggests that using other drugs or other approaches, it may be possible to restore PRH expression in prostate cancer cells without off-target effects and thereby inhibit tumour growth. Further work is required to explore this potential approach to cancer treatment.

## 5. Conclusions

PRH mRNA and protein levels are decreased in prostate cancer cells compared to normal cells, and in mouse models, the expression of PRH in prostate cancer cells inhibits cell proliferation and reduces tumour formation and tumour growth. We conclude that PRH acts as a tumour suppressor protein in prostate cancer cells.

## Figures and Tables

**Table 1:** Primers used for qRT-PCR.

Target mRNA		Sequence (5′- to -3′)
β-actin	Top	AAAGACCTGTACGCCAACAC
	Bottom	GTCATACTCCTGCTTGCTGAT
HHEX/PRH	Top	AAACCTCTACTCTGGAGCCC
	Bottom	GGTCTGGTCGTTGGAGAATC
TGFB2	Top	AGGAAAGGCGGGTAATGGAA
	Bottom	AAGGACTGCTGGGATGACAA
TGFBR2	Top	AAATGCTGGCTCTACACCCT
	Bottom	GGGGATGTTGGACAGGAAGA
CDH1	Top	GTAACGACGTTGCACCAACC
	Bottom	AGCCAGCTTCTTGAAGCGAT

**Table 2:** ChIP primers.

Target mRNA		Sequence (5′- to -3′)
Chromosome 18	Top	TTCAGTCTGGTGGTGGTGAACT
	Bottom	GCCTTGGGAAATCCATCTTTT
*CDH1* + 4.5 kb	Top	AGCCTAGTAACCACAGCTGT
	Bottom	TCAAGCAGCCAAACCTCAAC
*TGFB2*	Top	GGAACCTGTGCTGCTTTGTA
	Bottom	AGGTGTGGGTATGAACGGAA
*TGFBR2*	Top	TCAAAACTGTGTTCCTGGCT
	Bottom	AGTTGCAGCCTCAGATGACT

## Data Availability

All data will be made publicly available on publication.

## Supplementary Materials

The following supporting information can be downloaded at: https://www.mdpi.com/article/10.3390/cancers18142247/s1. Figure S1: Methylation of the *HHEX* gene 5′ CpG island in prostate cancer cells. Figure S2: Nuclear PRH protein staining in prostate adenocarcinoma samples. Figure S3: Effects of doxycycline on the viability of PC3 cells. Figure S4: Effects of doxycycline induction on PC3 PRH Lv cells. Figure S5: Effects of CX4945 on the viability of PC3 cells. Figure S6: Original Western blots. Figure S7: ChIP data.
