# Supplementary material for: Down-Regulation of Proline Rich Homeodomain/Haematopoietically Expressed Homeobox Expression in Prostate Cells Enables Tumour Initiation and Tumour Growth
Source: Cancers (Basel). 2026 Jul 14;18(14):2247. doi: 10.3390/cancers18142247 (PMC13406419; doi:10.3390/cancers18142247)

SUPPLEMENTARY FIGURES

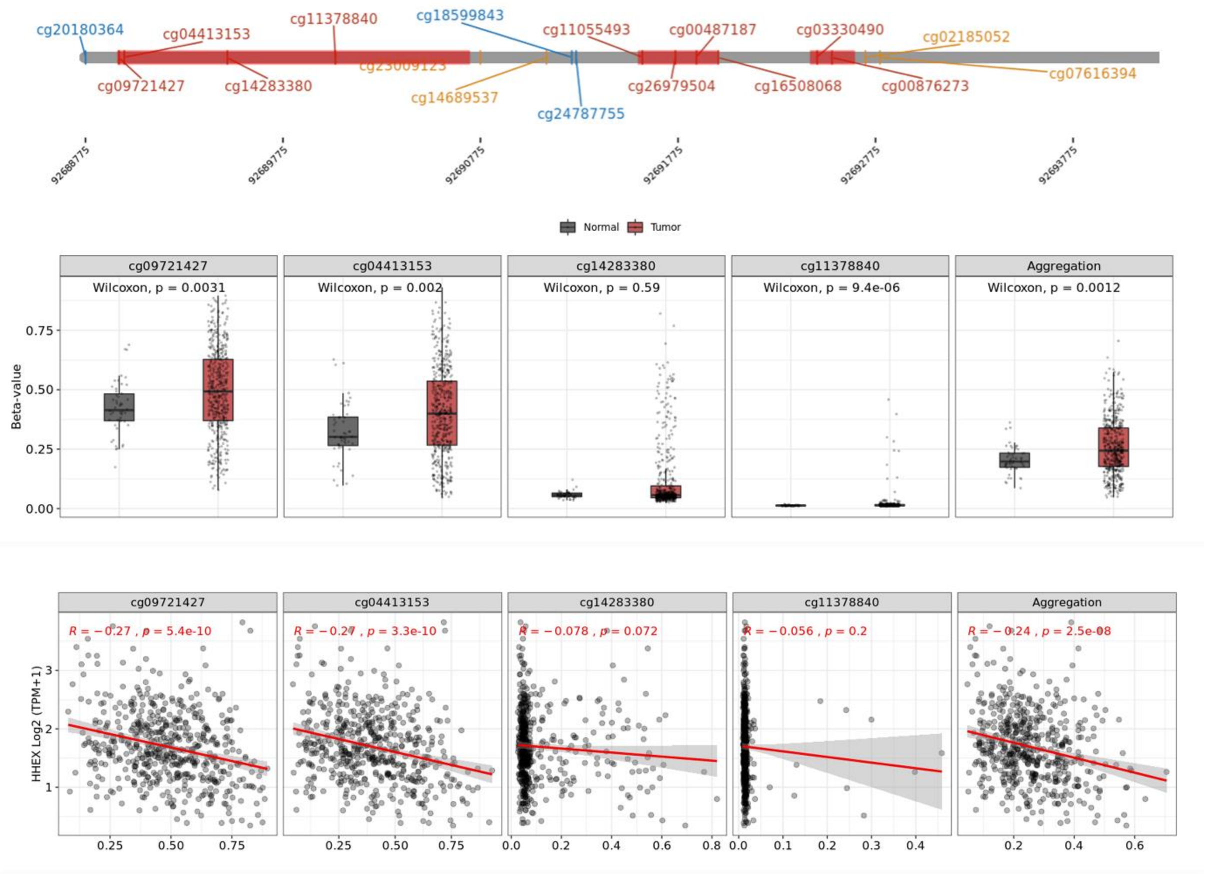

Figure S1 CpG methylation of the HHEX gene in prostate cancer cells. Top - CpG probe locations spanning the HHEX gene. Middle **-** CpG methylation data (β values) for individual CpG sequences in the *HHEX* gene 5′ CpG island produced from TCGA data using SMART [45]. Bottom - scatter plots showing the correlation of CpG methylation data (β values) at the locations in the 5′ CpG island and *HHEX* mRNA levels.

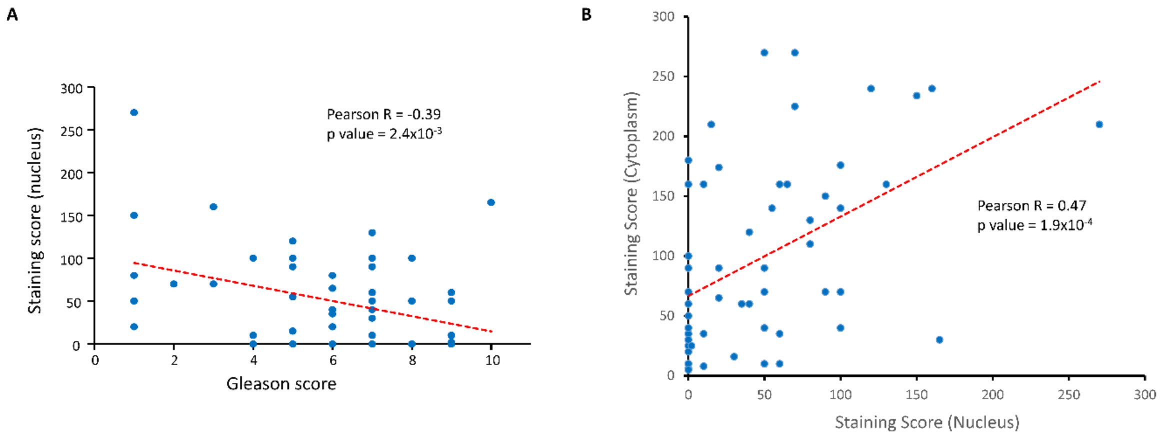

Figure S2 Nuclear PRH protein staining in prostate adenocarcinoma samples. (A) A scatter plot of nuclear PRH immunostaining score versus Gleason score. (B) A scatter plot of cytoplasmic PRH immunostaining score versus nuclear PRH immunostaining score.

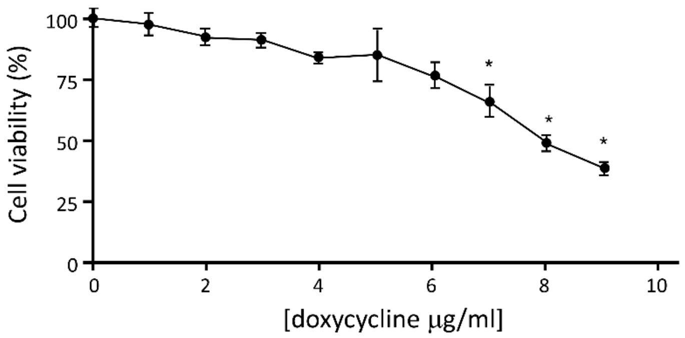

Figure S3 Effects of doxycycline on the viability of PC3 cells. PC3 cells were treated with vehicle or increasing amounts of doxycycline for 24 hours and cell viability was then determined using an MTT assay. Mean and SEM. One-way ANOVA, * = $p < 0.05$.

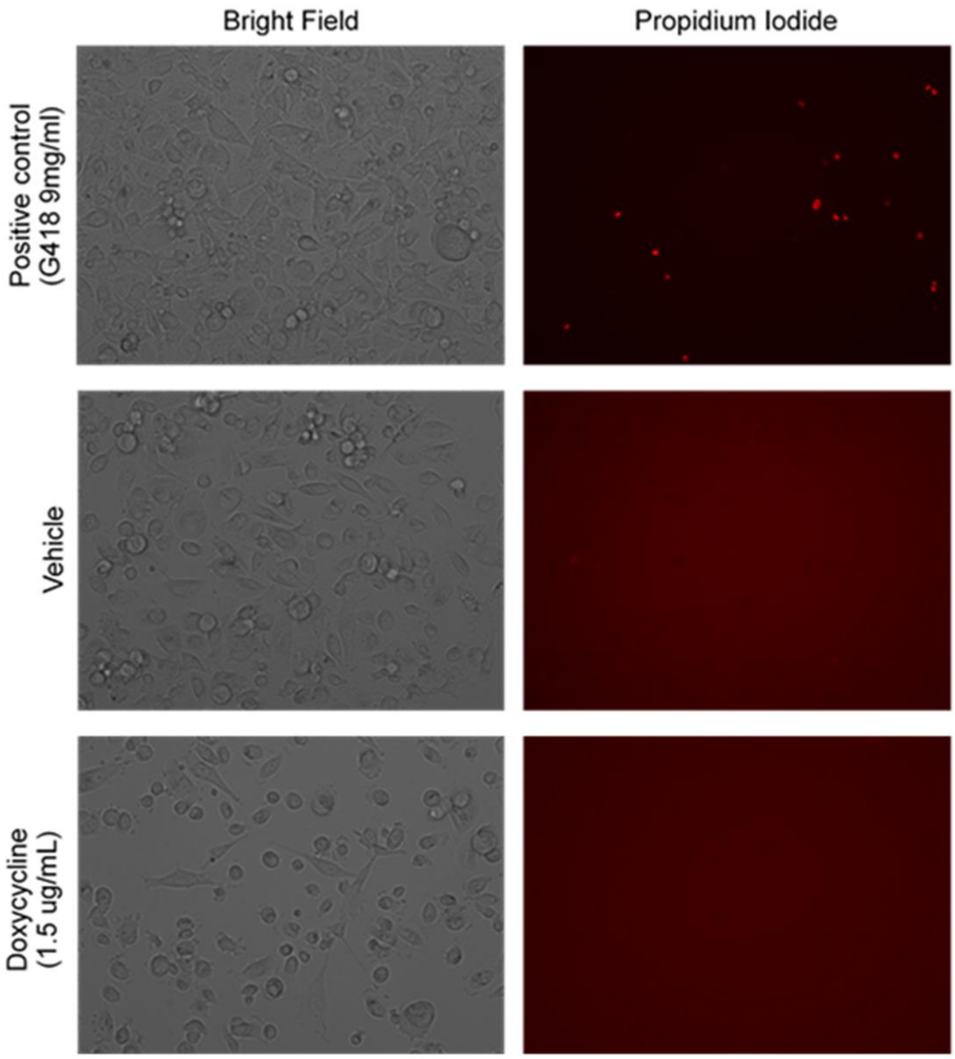

Figure S4 Effects of doxycycline on PC3 PRH Lv cells. PC3 PRH Lv cells with 9mg/ml G418 for 24 hours as a positive control, 1.5 ug/mL of doxycycline for 7 days, or vehicle control for 7 days. The cells were then stained with propidium iodide and imaged using a Leica DMI 6000 B microscope. The positive control presented an average of 15 cells/field stained with propidium iodide.

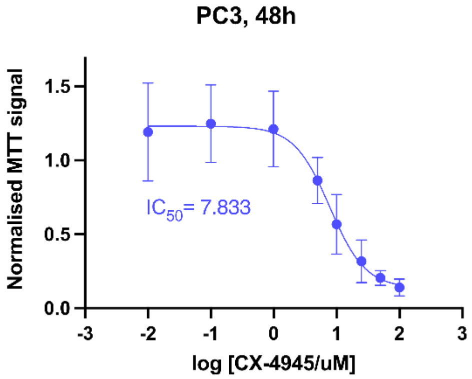

Figure S5 Effects of CX4945 on the viability of PC3 cells. PC3 cells were treated with increasing concentrations of CX4945 for 48 hours and cell viability was then measured using an MMT assay. Mean and SEM. N=5 biological repeats.

Figure S6 Original western blots

(A) Figure 2A

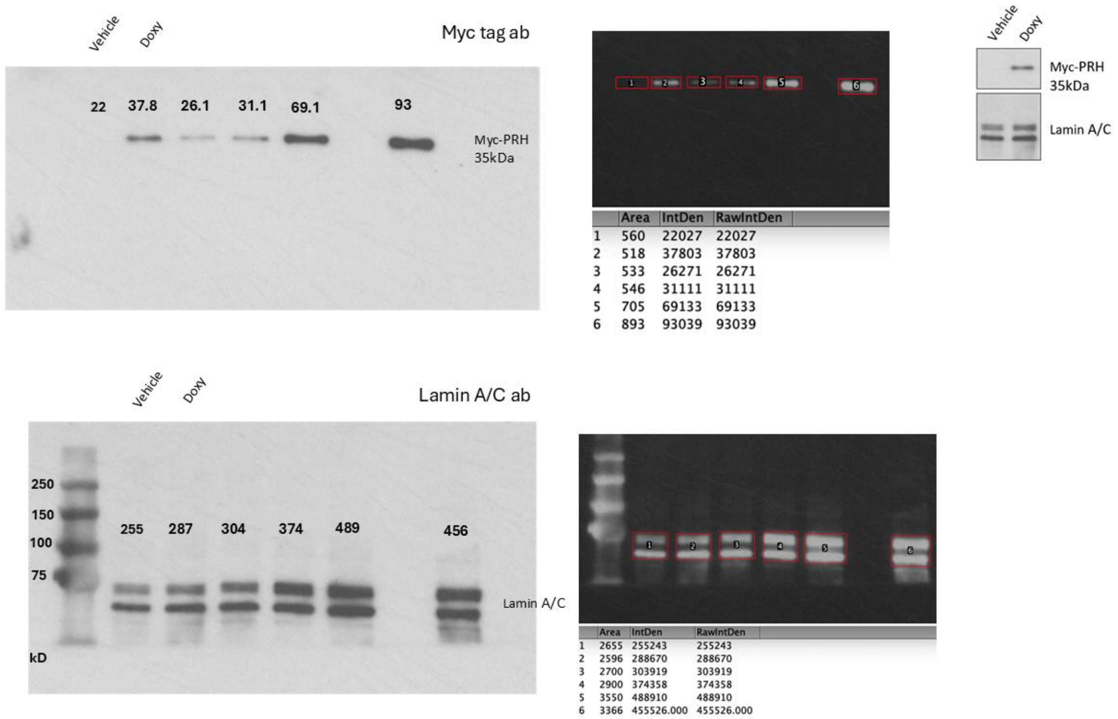

Figure S6 Original western blots
(B) Figure 4C

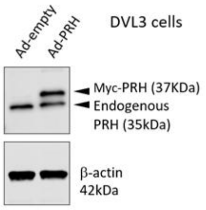

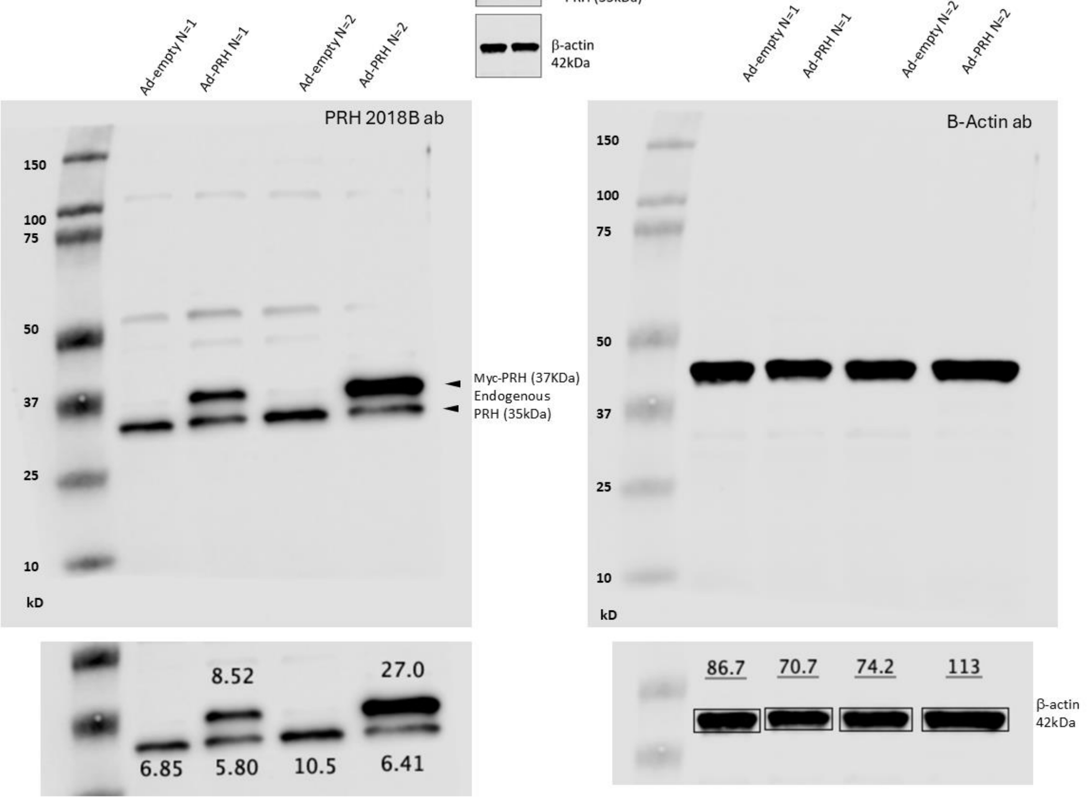

Figure S6 Original western blots

(C) Figure 5A and 5C

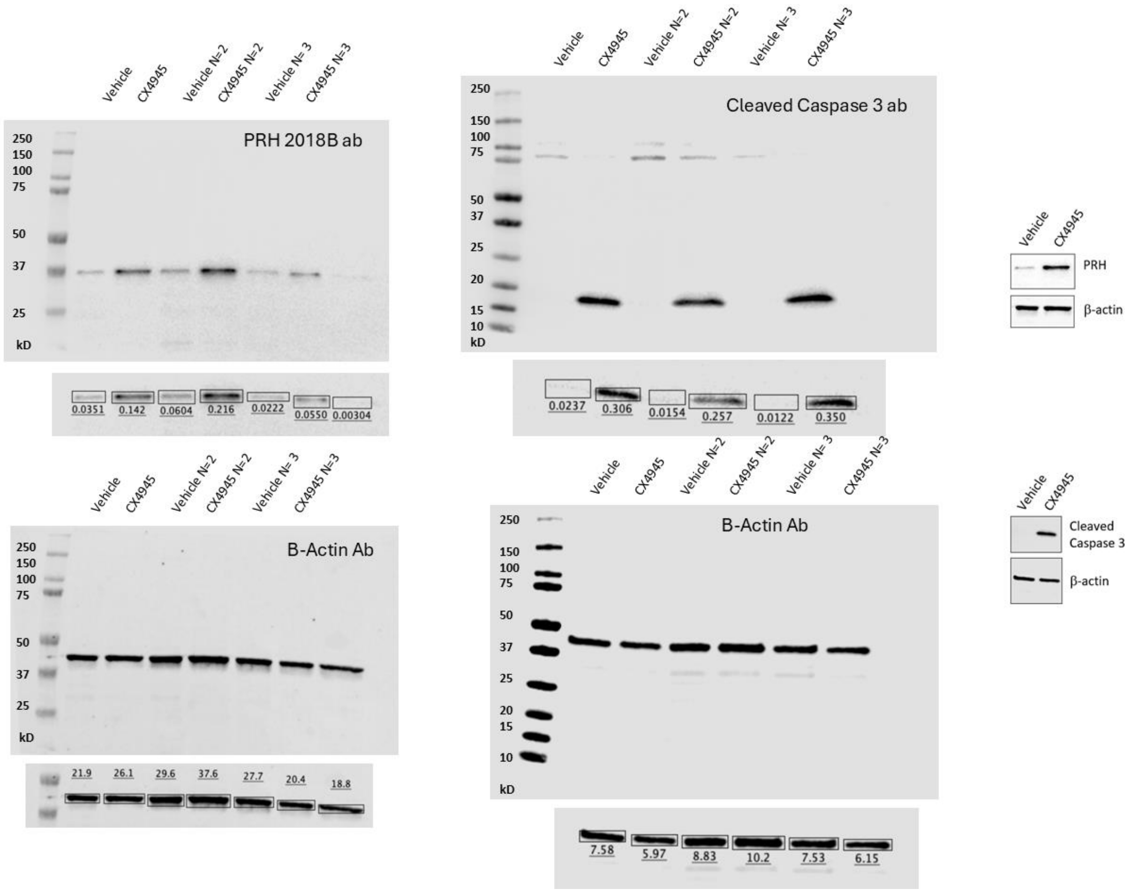

Figure S7 ChIP data

**TGFBR1**	DE1 (IGG ChIP)	DE1 (Myc-Tag ChIP)	Myc-PRH (IGG ChIP)	Myc-PRH (Myc-tag ChIP)
Average	0.013268369	0.005320317	0.043185946	0.205263567
Standard Error	0.005502748	0.001553944	0.01613824	0.022020319
**TGFBR2 -11K**	DE1 (IGG ChIP)	DE1 (Myc-Tag ChIP)	Myc-PRH (IGG ChIP)	Myc-PRH (Myc-tag ChIP)
Average	0.0157077	0.012756969	0.04813738	0.1332804
Standard Error	0.002597052	0.003953435	0.009855697	0.012771574
**TGF2 +54K**	DE1 (IGG ChIP)	DE1 (Myc-Tag ChIP)	Myc-PRH (IGG ChIP)	Myc-PRH (Myc-tag ChIP)
Average	0.020076573	0.02128703	0.04646233	0.2033593
Standard Error	0.001788346	0.004473879	0.009337501	0.038529494
**CDH1 +4.5**	DE1 (IGG ChIP)	DE1 (Myc-Tag ChIP)	Myc-PRH (IGG ChIP)	Myc-PRH (Myc-tag ChIP)
Average	0.00704633	0.015608117	0.020266	0.1207474
Standard Error	0.001158707	0.002770334	0.003816519	0.006348169
**CDH1 -4.5K**	DE1 (IGG ChIP)	DE1 (Myc-Tag ChIP)	Myc-PRH (IGG ChIP)	Myc-PRH (Myc-tag ChIP)
Average	0.017000223	0.006673117	0.025829593	0.074084423
Standard Error	0.001617948	0.001924939	0.00561172	0.004074177
**CH18**	DE1 (IGG ChIP)	DE1 (Myc-Tag ChIP)	Myc-PRH (IGG ChIP)	Myc-PRH (Myc-tag ChIP)
Average	0.006509377	0.02640693	0.00848645	0.018821677
Standard Error	0.002540768	0.001190768	0.00370639	0.003503533
	DE1 = Ad Empty			
	Myc-PRH = Ad PRH			
	N=3			

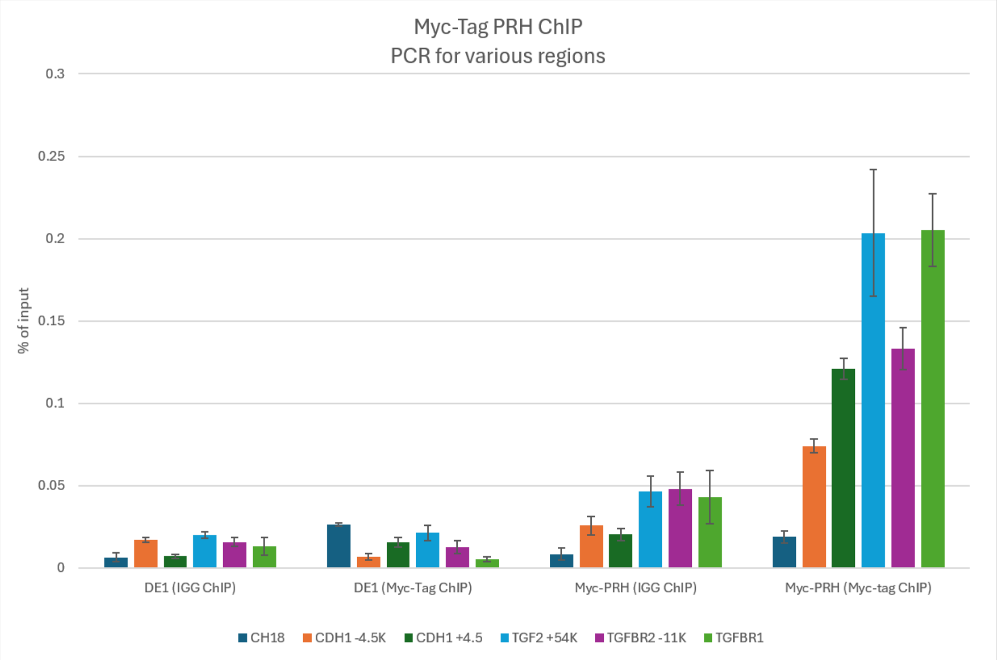

Supplement: Supplementary file 1 [file cancers-18-02247-s001.zip › SUPPLEMENTARY FIGURES.pdf]
